# Comparing Face-to-Face and Online Data Collection Methods in Preterm and Full-Term Children: An Exploratory Study

**DOI:** 10.3389/fpsyg.2021.733192

**Published:** 2021-10-28

**Authors:** Paige M. Nelson, Francesca Scheiber, Haley M. Laughlin, Ö. Ece Demir-Lira

**Affiliations:** ^1^Department of Psychological and Brain Sciences, The University of Iowa, Iowa City, IA, United States; ^2^Delta Center, The University of Iowa, Iowa City, IA, United States; ^3^Iowa Neuroscience Institute, The University of Iowa, Iowa City, IA, United States

**Keywords:** COVID-19, in-person data collection, online data collection, children, prematurity, neurocognitive assessment, developmental psychology

## Abstract

The COVID-19 pandemic has transformed the landscape for children’s daily lives and the landscape for developmental psychology research. Pandemic-related restrictions have also significantly disrupted the traditional face-to-face methods with which developmental scientists produce research. Over the past year, developmental scientists have published on the best practices for online data collection methods; however, existing studies do not provide empirical evidence comparing online methods to face-to-face methods. In this study, we tested feasibility of online methods by examining performance on a battery of standardized and experimental cognitive assessments in a combined sample of 4- to 5-year-old preterm and full-term children, some of whom completed the battery face-to-face, and some of whom completed the battery online. First, we asked how children’s performance differs between face-to-face and online format on tasks related to verbal comprehension, fluid reasoning, visual spatial, working memory, attention and executive functioning, social perception, and numerical skills. Out of eight tasks, we did not find reliable differences on five of them. Second, we explored the role of parent involvement in children’s performance in the online format. We did not find a significant effect of parent involvement on children’s performance. Exploratory analyses showed that the role of format did not vary for children at risk, specifically children born preterm. Our findings contribute to the growing body of literature examining differences and similarities across various data collection methods, as well as literature surrounding online data collection for continuing developmental psychology research.

## Introduction

The COVID-19 pandemic has transformed the landscape for children’s daily lives and the landscape for developmental psychology research. Schools across the world have restructured and developed online learning curriculums. Around 214 million children are estimated to have missed more than three quarters of in-person education in 2020 (United Nations Children’s Fund, 2021). More than 90% of children in the United States are estimated to have received some form of distance learning during COVID-19 (Bureau, 2021). Likewise, the provision of community and social services has suffered, despite being needed now more than ever (Tsega et al., 2020; EducationData, 2021). Pandemic-related restrictions have also significantly disrupted the traditional methods in which developmental scientists produce research—that is, in-person studies requiring face-to-face interactions (Garrisi et al., 2020). Given the likely continued role of online assessments in research and clinical services, it is important to understand both the differences and similarities between children’s performance in face-to-face and online settings. The direct and long-term effects of online measurement of children’s performance remain largely unknown. The goal of the current paper is to add to this growing body of literature by testing the feasibility of online data collection methods and comparing 4- to 5-year-old preschoolers’ performance face-to-face vs. online on a wide variety of standardized and experimental cognitive assessments.

Over the past few years, developmental scientists have published on the best practices for online data collection methods (Frank et al., 2016; Garrisi et al., 2020; Lourenco and Tasimi, 2020; Manning et al., 2020; Nussenbaum et al., 2020; Rhodes et al., 2020; Sheskin et al., 2020; Morini and Blair, 2021; Su and Ceci, 2021). These studies agree that while online data collection methods are still in their infancy, online measurements have become a promising platform for developmental psychology research. However, only a few studies provide empirical evidence comparing online methods to face-to-face methods. For example, Morini and Blair (2021) examined the feasibility of collecting remote eye-gaze data with children. They compared their online sample to a previously collected face-to-face sample, during which they found their online data collection methods to be reliable and sufficient in conducting developmental language research (Morini and Blair, 2021).

An emerging body of work also focuses on the reliability and validity of online data collection methods. For example, Manning et al. (2020) examined the feasibility, reliability, and validity of child language samples drawn from recorded parent-child interactions via video chat. They found child language samples (i.e., key speech and language measures) collected via video chat vs. face-to-face laboratory video recordings to be comparable (Manning et al., 2020). So far, studies have focused on narrow aspects of children’s performance, thus it is important to examine children’s performance on a wide array of standardized and experimental measures assessing multiple cognitive domains to gain a more complete view of face-to-face vs. online assessments. Our primary goal is to examine how children’s performance in verbal comprehension, fluid reasoning, visual spatial, working memory, attention and executive functioning, social perception, and numerical tasks differ between a lab based, face-to-face format, and an online format.

According to some, remote research has many benefits, including its ability to broaden sample diversity, as compared to face-to-face laboratory studies (Lourenco and Tasimi, 2020). For example, online methods might be more inclusive of groups who do not or cannot attend face-to-face research studies, including atypically developing children. Given the greater need for assessments and interventions for at-risk children in clinical settings, it is fundamental to better understand whether and how online interactions influence children with atypical developmental trajectories. Children born preterm (<37 weeks gestational age) fall into such an “at risk” group. Every year, close to 15 million children in the United States are born preterm (Wolke et al., 2019). Those who survive have an increased risk for death, disability, and delay (Centers for Disease Control and Prevention [CDC], 2020). Preterm-born children (PTB) fall behind term-born children (TB) on various measures of cognitive performance (Allotey et al., 2018; Brydges et al., 2018), with children born the earliest tending to have the worst outcomes (Bhutta et al., 2002; Snijders et al., 2020). Likewise, this gap in cognitive performance between PTB and TB children often persists throughout formal schooling. Better understanding how at-risk children perform in face-to-face vs. online formats will have implications for future assessment and intervention efforts. Thus, in the current study, we diversify our sample by including both PTB children and TB children.

Taken together, our goal is to contribute to the growing literature on establishing the reliability of online research methods by examining children’s performance on standardized and experimental cognitive assessments. We examine performance in a combined sample of 4- to 5-year-old TB and PTB children, some of whom completed the battery face-to-face and some of whom completed the battery online. We ask how children’s performance differs between face-to-face and online format on tasks related to verbal comprehension, fluid reasoning, visual spatial, working memory, attention and executive functioning, social perception, and numerical skills. We supplement our main research question with two exploratory analyses. Online data collections methods typically rely on parents, but how parent involvement during remote data collection influences children’s performance has yet to be explored. To address this question, we explore the role of parent involvement in children’s performance in the online format. How children’s performance differs between face-to-face and online format on tasks related to verbal comprehension, fluid reasoning, visual spatial, working memory, attention and executive functioning, social perception, and numerical skills as a function of a prematurity also has yet to be explored. To address this question as an exploratory aim, we examine the role of prematurity in children’s performance in both the face-to-face and online format.

## Methods

### Participants

Participants were 93 TB (≥37 weeks gestational age) and 38 PTB (<37 weeks gestational age), for a total of 131 children, who participated in an ongoing longitudinal study on the relations between preterm birth and neurodevelopment. Fifty-four TB children and 29 PTB completed the study face-to-face in a lab-based format. Thirty-nine TB children and nine PTB completed the study in an online format via Zoom video conferencing. Overall, 83 children (29 PTB) completed the study face-to-face in a lab-based format and 48 children (9 PTB) completed the study in an online format. This study was approved by the Institutional Review Board at our local university. We recruited parent-child dyads using the university hospital’s electronic health records, university mass emailing, social media, and word of mouth. Parent-child dyads were eligible for this study if the child was between the ages of 4 and 5 years old, was a native speaker of English, had normal or corrected-to-normal vision and hearing, had no history of a genetic syndrome or birth defect, and had no limitations (based on parental report) that would prevent them from completing paper/pencil tasks. For those who completed the study in an online format, it was also preferred that they had an electronic device (computer, laptop, tablet, or smart phone) with reliable internet. Parent-child dyads without an electronic device were mailed an Amazon Fire tablet that they could use to participate in the online sessions. We began enrollment for the face-to-face study in June 2019 and paused data collection in March 2020, due to the COVID-19 pandemic. In October 2020, we began the enrollment for the online study, for which we used Zoom video conferencing. The online data collection is ongoing; for the purposes of the current manuscript, we report on data collected through mid-June 2021.

Table 1 shows demographic characteristics for the face-to-face and online samples. Face-to-face and online parent-child dyads did not significantly differ in child age, gender, ethnicity, race, gestational age, birthweight, parent education, or household income. Children and parents were predominately White and from high-socioeconomic backgrounds, with an average household income of $115,648.71 and an average parent education corresponding to a college degree.

**TABLE 1:** Demographic information for face-to-face (*N* = 83) and online (*N* = 48) samples.

	Face-to-face	Online
	*M* (*SD*) or *n* (%)	*M* (*SD*) or *n* (%)
Child age (years)	4.77 (0.48)	5.15 (0.48)
**Child gender**		
Female	38 (46%)	25 (53%)
Child hispanic	7 (8%)	4 (9%)
Child white	80 (96%)	45 (94%)
Child premature	29 (35%)	9 (19%)
Birth weight (lbs, oz)	6.20 (2.42)	6.81 (1.71)
Household income (USD)	113233 (67708)	120204 (56229)
**Parent education**		
High school graduate	3 (4%)	2 (4%)
Some college credit	5 (6%)	6 (13%)
Associate’s degree	10 (12%)	4 (9%)
Bachelor’s degree	37 (45%)	10 (21%)
Professional degree	28 (34%)	25 (53%)
Parent age (years)	36.59 (4.87)	38.61 (13.04)
**Parent gender**		
Female	75 (91%)	43 (93%)
Parent hispanic	3 (4%)	1 (2%)
Parent white	77 (93%)	45 (94%)

### Procedure

For the face-to-face portion of the study, parent-child dyads attended a 3-h laboratory visit. During the laboratory visit, experimenters administered tasks to children, while parents completed questionnaires on a computer in another room. The face-to-face portion of the study included ten standardized neurocognitive assessments and four experimental tasks. The standardized neurocognitive assessments included six subtests from the Wechsler Preschool & Primary Scale of Intelligence, Fourth Edition (WPPSI-IV; Wechsler, 2012) (block design, bug search, matrix reasoning, information, similarities, and picture memory) and four subtests from the Developmental Neuropsychological Assessment, Second Edition (NEPSY-II; Brooks et al., 2009) (affect recognition, comprehension of instruction, statue, and theory of mind). The three experimental tasks included Give A Number, What’s on This Card, and Mental Rotation. Tasks were administered in blocks, and children took breaks in between each block.

For the online portion of the study, parent-child dyads participated in four 45–60-min sessions via Zoom. Using feedback from a focus group with local parents who expressed concern regarding possible screen fatigue, we structured the online portion of the study across four, shorter online sessions rather than one 3-h session. During the online sessions, children completed most the same standardized neurocognitive assessments, and parents completed the same online questionnaires. In the online portion of the study, parents were asked to complete the same questionnaires on their own time between session 1 and session 4. Four of the neurocognitive assessments (WPPSI-IV block design, give a number, NEPSY-II comprehension of instructions, and NEPSY-II theory of mind) that were administered face-to-face could not reliably be administered in an online format for reasons discussed below. The six remaining standardized neurocognitive assessments and two remaining experimental tasks were divided across four online Zoom sessions. Session 1 included WPPSI-IV matrix reasoning, information, and similarities. Session 2 included what’s on this card and mental rotation. Session 3 included NEPSY-II affect recognition and statue. Session 4 included WPPSI-IV picture memory. The order of the tasks was the same in both the face-to-face and online sessions, and tasks were administered by the same research assistants.

For the online portion of the study, parents scheduled their four sessions via Calendly (an online scheduling tool), email, or phone. Once parents scheduled their sessions, experimenters provided parent-child dyads with information to prepare them for their first session. This included information on preparing devices and information on dos and don’ts for the four sessions. For example, experimenters stressed the importance of not providing aid or input during the neurocognitive assessments. This also included a link to the informed consent document if the parent did not fill it out prior. The day of session 1, experimenters emailed parents a secured Zoom video conferencing link, for which they were able to attend without needing a Zoom account. Once parent-child dyads logged onto the session, experimenters guided parents on positioning the camera if necessary. With parental consent, all online sessions were recorded through Zoom. The same procedures were followed for session 2 through session 4. Further information on task set up is in Table 2.

**TABLE 2:** Task descriptions for face-to-face and online procedures.

**Task**	**Included face-to-face?**	**Included online?**	**Timing/response type for online**	**Face-to-face procedure**	**Online procedure**
**Verbal comprehension**					
WPPSI-IV information	✓	✓	Untimed / verbal response	For questions involving pictures, experimenters presented children with visual stimuli via a testing binder, and flipped the pages when children were ready to move items. Children provided their response by pointing. For questions involving verbal response, experimenters read questions aloud, and children responded verbally.	For questions involving pictures, experimenters presented children with visual stimuli via Microsoft PowerPoint; experimenters shared screen and advanced slides when children were ready to move items. Children provided their response by ‘stamping’ via Zoom’s remote-control feature, on the experimenter’s screen using a computer mouse or trackpad. Parents were asked to assist, during which they helped their child ‘stamp’ their response. For questions involving verbal response, participants were administered the Information subtest online using the same method as in-person.
WPPSI-IV similarities	✓	✓	Untimed / verbal response	For questions involving pictures, experimenters presented children with visual stimuli via a testing binder, and flipped the pages when children were ready to move items. Children provided their response by pointing. For questions involving verbal response, experimenters read questions aloud, and children responded verbally.	For questions involving pictures, experimenters presented children with visual stimuli via Microsoft PowerPoint; experimenters shared screen and advanced slides when children were ready to move items. Children provided their response by ‘stamping’ via Zoom’s remote-control feature, on the experimenter’s screen using a computer mouse or trackpad. Parents were asked to assist, during which they helped their child ‘stamp’ their response. For questions involving verbal response, participants were administered the Similarities subtest online using the same method as in-person.
**Language**					
NEPSY-II comprehension of instructions	✓		Untimed / response stamp	Experimenters presented children with visual stimuli via a testing binder, during which experimenters gave verbal instructions that increased in complexity and could not be repeated. Children provided their response by pointing.	
**Visuospatial**					
WPPSI-IV block design	✓			Experimenters presented children with blocks of various colors and patterns and asked children to model 3-D block patterns of increased complexity	
Mental rotation	✓	✓	Untimed / response stamp	Experimenters presented children with visual stimuli via a testing binder and flipped the pages when children were ready to move items. Children provided their response by pointing.	Experimenters presented children with visual stimuli via Microsoft PowerPoint; experimenters shared screen and advanced slides when children were ready to move items. Children provided their response by ‘stamping’ via Zoom’s remote-control feature, on the experimenter’s screen using a computer mouse or trackpad. Parents were asked to assist, during which they helped their child ‘stamp’ their response.
**Fluid reasoning**					
WPPSI-IV matrix reasoning	✓	✓	Untimed / response stamp	Experimenters presented children with visual stimuli via a testing binder and flipped the pages when children were ready to move items. Children provided their response by pointing.	Experimenters presented children with visual stimuli via Microsoft PowerPoint; experimenters shared screen and advanced slides when children were ready to move items. Children provided their response by ‘stamping’ via Zoom’s remote-control feature, on the experimenter’s screen using a computer mouse or trackpad. Parents were asked to assist, during which they helped their child ‘stamp’ their response.
**Working memory**					
WPPSI-IV picture memory	✓	✓	Timed / response stamp	Experimenters presented children with visual stimuli via a testing binder and flipped the pages when children were ready to move items. Children provided their response by pointing.	Experimenters presented children with visual stimuli via Microsoft PowerPoint; experimenters shared screen and advanced slides when children were ready to move items. Children provided their response by ‘stamping’ via Zoom’s remote-control feature, on the experimenter’s screen using a computer mouse or trackpad. Parents were asked to assist, during which they helped their child ‘stamp’ their response.
**Attention and** **executive functioning**					
NEPSY-II statue	✓	✓	Timed	Experimenters administered the Statue subtest online using the same method as in-person, with the exception that experimenters often asked parents to help orient their child in the position of the camera. Otherwise, parents were not required to assist on the Statue subtest.	
**Social perception**					
NEPSY-II affect recognition	✓	✓	Untimed / response stamp	Experimenters presented children with visual stimuli via a testing binder and flipped the pages when children were ready to move items. Children provided their response by pointing.	Experimenters presented children with visual stimuli via Microsoft PowerPoint; experimenters shared screen and advanced slides when children were ready to move items. Children provided their response by ‘stamping’ via Zoom’s remote-control feature, on the experimenter’s screen using a computer mouse or trackpad. Parents were asked to assist, during which they helped their child ‘stamp’ their response.
NEPSY-II theory of mind	✓		Untimed / verbal response / response stamp	For the Verbal task and contextual tasks, experimenters presented children with visual stimuli via a testing binder and flipped the pages when children were ready to move items. For the Contextual task, children provided their response by pointing.	
**Numerical**					
What’s on this card	✓	✓	Untimed / verbal response	Experimenters presented children with visual stimuli via a testing binder and flipped the pages when children were ready to move items. Children provided their response by responding verbally.	Experimenters presented children with visual stimuli via Microsoft PowerPoint; experimenters shared screen and advanced slides when children were ready to move items. Children provided their response by responding verbally. Parents were not required to assist on the WOC task.
Give a number	✓			Experimenters presented children with a pile of fifteen plastic fish, during which experimenters asked children to place a certain number of fish (i.e., 1–9) into a fishbowl.	
**Processing speed**					
WPPSI-IV bug search	✓		Timed / response stamp	Experimenters presented children with visual stimuli via a testing packet and flipped the pages when children were ready to move items. Experimenters asked the children to match various kinds of bugs to one another from an assortment of response options using a child-friendly ink dauber, within one minute and 15 seconds.	

### Measures Administered Both Face-to-Face and Online

Experimenters administered the following measures. It should be noted that publishers of standardized neurocognitive assessments did not provide the online materials; we adapted the materials for online administration for research purposes. This is true for all WPPSI-IV and NEPSY-II materials. The WPPSI-IV and NEPSY-II have been shown to have strong reliability and validity when measured in a face-to-face format (Brooks et al., 2009; Wechsler, 2012). We also measured internal consistency using Cronbach’s alpha in our two experimental tasks. Reliability across face-to-face and online participants on What’s on This Card was good (a > 0.75). Reliability across face-to-face participants on Mental Rotation was acceptable (*a* = 0.50), and across online participants, reliability was good (*a* = 0.74). Please see Table 2 for further details on the tasks referenced below.

#### Verbal Comprehension

##### WPPSI-IV information

Experimenters administered the Information subtest, part of the WPPSI-IV Verbal Comprehension Index (VCI). The VCI measures a child’s acquired knowledge, verbal reasoning, and comprehension skills. The Information subtest uses both visual and verbal stimuli to assess children’s acquired knowledge (e.g., “what do people use to stay dry in the rain?”) (Wechsler, 2012).

##### WPPSI-IV similarities

Experimenters administered the Similarities subtest, part of the WPPSI-IV VCI. The Similarities subtest asks children, using Picture Tasks and Verbal Tasks, to describe how two words that share a common characteristic are related to one another (e.g., “red and yellow are both…”) (Wechsler, 2012).

#### Fluid Reasoning

##### WPPSI-IV matrix reasoning

Experimenters administered the Matrix Reasoning (WPPSI-MR) subtest, part of the WPPSI-IV Fluid Reasoning Index (FRI). WPPSI-MR measures visual processing and spatial perception by asking children to select a missing portion from a matrix (Wechsler, 2012).

#### Visual Spatial

##### Mental rotation

Experimenters administered a shortened version of the Children’s Mental Transformation Task (CMTT, Levine et al., 1999). The Children’s Mental Transformation Task is a non-verbal spatial task, during which children are presented with four shapes and two halves of a 2D shape and asked to select the shape that the two halves would make if they were put together.

#### Working Memory

##### WPPSI-IV picture memory

Experimenters administered the Picture Memory (WPPSI-PM) subtest, part of the WPPSI-IV Working Memory Index (WMI). WPPSI-PM measures a child’s working memory by asking children to look at pictures of increasingly complex quantities, for three seconds, before asking children to point to those they viewed on a response page (Wechsler, 2012).

#### Attention and Executive Functioning

##### NEPSY-II statue

Experimenters administered the Statue subtest, part of the NEPSY-II Attention and Executive Functioning domain, which measures a child’s motor persistence and inhibition (Brooks et al., 2009). Experimenters asked children to remain as still as possible, during which experimenters deducted points if children opened their eyes, made drastic body movements, and/or spoke.

#### Social Perception

##### NEPSY-II affect recognition

Experimenters administered the affect recognition (AR) subtest, part of the NEPSY-II Social Perception domain. The AR subtest measures a child’s ability to recognize affect (Brooks et al., 2009). In four different tasks, experimenters showed children variations of affect from photographs of children’s faces, during which experimenters’ assessed children’ ability to recognize affect between children in each task (Brooks et al., 2009).

#### Numerical

##### What’s on this card

Experimenters administered a What’s on This Card task, during which experimenters asked children to vocalize what they saw on twelve different cards. For example, experimenters showed a card with three soccer balls and asked children “what is on this card?” Experimenters scored the total amount of cards the participant responded correctly to, out of a total of seventeen.

### Measures Administered Face-To-Face but Not Online

We were unable to move several face-to-face measures to an online format. Some tasks were excluded because we were not able to provide the necessary materials to each participant. For example, we could not administer the WPPSI-IV block design (BD) subtest, part of the WPPSI-IV visual spatial index (VSI), in an online format because we were unable to supply the standardized materials (i.e., blocks, assessment binder, and stop-watch) to every participant. Likewise, we could not administer WPPSI-IV bug search (BS) subtest, part of the WPPSI-IV processing speed index (PSI), and an experimental numerical task, give a number task, due to the same reason.

Other tasks were excluded because we were concerned about administering these tasks in a standardized fashion across various electronic devices (i.e., laptop vs. tablet) and internet reliabilities. For example, we chose to not administer the NEPSY-II comprehension of instructions (CI) subtest, part of the NEPSY-II Language domain, due to being unable to repeat the verbal instructions to the child in case a problem with internet connection occured. We chose to not administer the NEPSY-II theory of mind (TM) subtest, part of the NEPSY-II Social Perception domain, due to the same reason.

### Parental Involvement Coding

For online sessions only, we categorized parental involvement into two categories: (1) parent absent or quiet and (2) parent present but with minimal involvement, including directing attention or rewording the instructions. If the parent was present with significant, more than minimal, involvement, the child’s score from that task was excluded from the analyses below. Examples of significant involvement included parents providing strategies relevant to the task that would significantly influence child performance or parents simply providing the answer. This coding was completed after the sessions were done using the video recordings of the interaction.

### Analytic Plan

We examined whether children’s performance varied as a function of format. A sequence of multiple linear regressions were run using the *lm* function in the *stats* package (R Core Team, 2013) to determine whether children in the online condition performed differently than children administered the battery face-to-face. Format was dummy coded with face-to-face condition used as the reference group. In predicting performance on WPPSI-IV and NEPSY-II, we controlled for parent education only because we used participant’s scaled scores adjusted for age at testing. In predicting performance on What’s On This Card and Mental Rotation, we controlled for parent education and child age at testing. For exploratory purposes, we also examined if child performance differed in the face-to-face and online format as a function of prematurity (TB vs. PTB) by later adding the interaction component between prematurity and format to the sequence of multiple linear regressions used to answer research question 1. Finally, again for exploratory analyses, we examined if child performance at a given session varied as a function of parental involvement in the session during which task was administered using *t*-tests with involvement as two categories (no involvement vs. minimal involvement).

## Results

### How Do Children’s Performance in Verbal Comprehension, Fluid Reasoning, Visual Spatial, Working Memory, Attention and Executive Functioning, Social Perception, and Numerical Skills Differ From Face-to-Face to Online Format?

**FIGURE 1:**
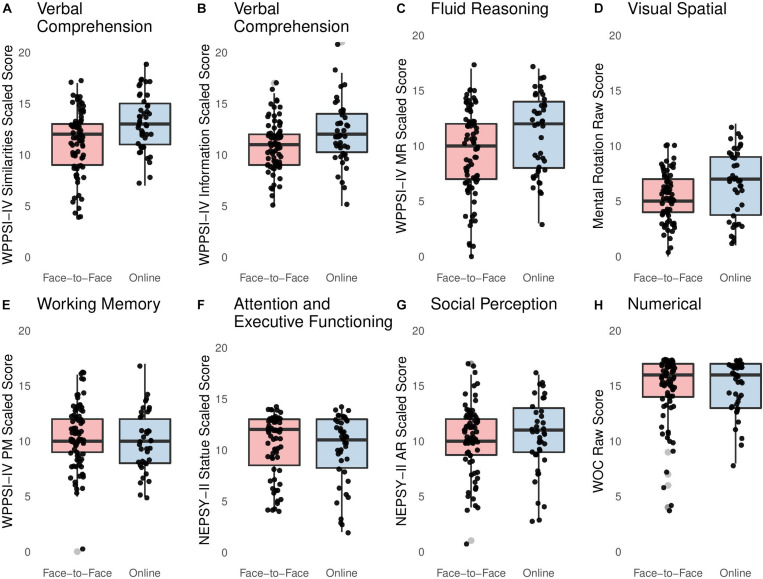
Box plots representing distribution of scores in both formats: **(A)** WPPSI-IV Similarities Scaled Score, **(B)** WPPSI-IV Information Scaled Score, **(C)** WPPSI-IV MR Scaled Score, **(D)** Mental Rotation Raw Score, **(E)** WPPSI-IV PM Scaled Score, **(F)** NEPSY-II Statue Scaled Score, **(G)** NEPSY-II AR Scaled Score, and **(H)** WOC Raw Score.

Figure 1 and Table 3 represent children’s performance on several measures, both in the face-to-face and in the online study. Face-to-face and online samples differed significantly from each other on the following tasks: WPPSI-IV information, WPPSI-IV similarities, and WPPSI-IV MR. Scores on all three subtests were reliably lower in the face-to-face sample than in the online sample, when controlling for parent education.

**TABLE 3:** Results of mean scores captured in both formats, participants included and excluded, and regression analyses comparing format controlling for parent education and child age at testing.

	Face-to-face			Online			Regression			
	*M* (*SD*)	*n*	*Ex.*	*M* (*SD*)	*n*	*Ex.*	Beta estimate (SE)	*t value*	*p*	*Adjusted R^2^*
**Verbal comprehension**										
WPPSI-IV similarities	11.22 (3.15)	76	7	13.18 (2.68)	45	3	1.86 (0.56)	*t*(117) = 3.33	<0.01**	0.10
WPPSI-IV information	10.92 (2.41)	77	6	12.35 (3.29)	46	2	1.46 (0.50)	*t(*119) = 2.94	<0.01**	0.14
**Fluid reasoning**										
WPPSI-IV matrix reasoning	9.28 (3.93)	78	5	11.02 (3.54)	42	6	1.83 (0.73)	*t*(116) = 2.50	0.01*	0.04
**Visual spatial**										
Mental rotation	5.37 (2.22)	70	13	6.55 (3.07)	40	8	0.20 (0.46)	*t*(105) = 0.43	0.67	0.31
**Working memory**										
WPPSI-IV picture memory	10.48 (2.86)	79	4	10.03 (2.78)	38	10	−0.47 (0.56)	*t*(114) = −0.83	0.41	−0.01
**Attention and executive functioning**										
NEPSY-II statue	10.38 (3.09)	63	20	10.05 (3.41)	42	6	−0.36 (0.65)	*t*(101) = −0.55	0.58	−0.01
**Social perception**										
NEPSY-II affect recognition	10.07 (3.12)	76	7	10.33 (3.35)	40	8	0.30 (0.61)	*t*(112) = 0.50	0.62	0.03
**Numerical**										
What’s on this card	14.65 (3.03)	78	5	14.93 (2.36)	41	7	−0.33 (0.57)	*t*(114) = −0.58	0.56	0.06

Statistics represent beta estimate, standard error, *T* Value, and *P* value for the Effect of Format. Ex., excluded. *p < 0.05; **p < 0.01.

Scores on the following standardized neurocognitive assessments did not differ between children who participated face-to-face and children who participated online, when controlling for parent education: WPPSI-IV PM, NEPSY-II AR, and NEPSY-II statue. Scores on the following experimental tasks did not differ between children who participated face-to-face and children who participated online, when controlling for parent education and age at testing: mental rotation and what’s on this card.

It could be the case that the lowest performing children were unable to perform the tasks online, resulting in experimenters dropping their scores. To test this possibility, we conducted a follow-up analysis, in which we compared whether the number of children whose data were dropped in the face-to-face vs. online study differed from each other. Data were excluded for the following reasons: significant parent involvement, child fatigue, and experimenter error. The number of children whose data were included vs. excluded from the analysis are reported in Table 3 and were compared across the two formats using Chi-Square analyses. None of the comparisons reached statistical significance (all *p*’s > 0.05), suggesting that number of children whose data were excluded due to reasons stated above did not vary across formats.

### What Is the Role of Parental Involvement in Children’s Performance in the Online Format?

Finally, for the children who participated in the online format only, we examined if parent involvement played a role. To reduce the number of analyses we ran, we only conducted these exploratory analyses on the three WPPSI-IV subtests on which children performed better in the online format as compared to face-to-face format. For children whose data were included in the first session, twenty-five parents were not involved and 22 were minimally involved. *t*-test analyses comparing children of parents who were not involved vs. those who were minimally involved did not reveal any significant differences on WPPSI-IV similarities, *t*(42) = 0.08, *p* = 0.94, WPPSI-IV information, *t*(43) = 2.02, *p* = 0.36, or on WPPSI-IV MR, *t*(39) = 1.23, *p* = 0.23.

### How Do Children’s Performance in Verbal Comprehension, Fluid Reasoning, Visual Spatial, Working Memory, Attention and Executive Functioning, Social Perception, and Numerical Skills Differ From Face-to-Face to Online Format as a Function of Prematurity?

Scores on all standardized neurocognitive assessments did not differ between children who participated face-to-face and children who participated online as a function of prematurity, except on WPPSI-IV MR. A reliable bivariate interaction emerged between format and prematurity on WPPSI-IV MR, *t*(114) = −2.22, *p* < *0.05.* PTB children performed lower than TB children in the online format, as compared to children in the face-to-face format.

## Discussion

The COVID-19 pandemic has catalyzed an increasing interest in the best practices for online data collection methods in developmental science. As COVID-19 restrictions persist, remote methods will be paramount to developmental science research. Here, we aimed to contribute to the discussions on online data collection methods. Specifically, we asked whether children who participated in study visits face-to-face and children who participated in study visits online performed differently on both standardized and experimental measures. We examined this question in both typically developing, term-born (TB) children, and in at-risk, preterm-born (PTB) children. We also explored whether parental involvement in the online format related to children’s performance.

The finding that children’s performance did not vary across the two formats for most of the measures that we administered provides support for the utilization of online data collection methods in developmental science. Here, we provide empirical evidence suggesting that children’s performance was not significantly influenced by format on a wide range of cognitive assessments, both standardized and experimental. Our results may help alleviate some of the concerns that researchers have raised about online data collection methods. First, researchers have expressed concern about having less control over the testing environment and having a higher number of distractions, which might lead to a greater portion of data being excluded from online formats. However, we examined this hypothesis, and this was not the case in our sample. It is important to highlight that, in both formats, trained clinical science graduate students audited study visits and excluded data that was thought to be an inaccurate representation of the child’s performance. For example, if it was clear that the child was not answering questions due to shyness, that child’s data was excluded. Second, there has been concern about differences in sample characteristics between samples that participate face-to-face and samples that participate online, including concerns about differences in demographic characteristics (e.g., parental education) and in health characteristics (e.g., children with special health needs). In our sample, group comparisons did not reveal any significant sociodemographic differences between the two groups. It should be noted that the average income and average education of our sample were generally high, so differences might emerge in a more socioeconomically diverse sample.

Moreover, we were able to recruit PTB children, who are at-risk for academic challenges. Although we were not sufficiently powered to include analyses on interactions between prematurity and format, we conducted exploratory analyses examining such interactions. Reliable bivariate interactions between format and prematurity did not emerge on any of the tests, except for WPPSI-IV MR; PTB children performed lower than TB children in the online format, but not face-to-face format. Thus, overall, format did not appear to greatly undermine preterm children’s performance. Future studies that are sufficiently powered should examine interactions between format and risk factors.

While children’s performance did not vary by format on most of the assessments, their scores on WPPSI-IV information, similarities, and matrix reasoning did. Average scores on these three subtests were lower for those who participated in the face-to-face format. This finding is inconsistent with findings from a previous study, suggesting equivalence in online and face-to-face scores on information, similarities, and matrix reasoning (Wright, 2020). However, this study included older children and leveraged proctors instead of parents. Thus, it is possible that parents played a role in our findings. However, our analyses showed that children who had more parental involvement and children who had no parental involvement did not differ on information, similarities or matrix reasoning. Another possibility is that the online format lent itself to performing better, due to children participating in the study in the comfort their own homes. During face-to-face study visits, children completed testing in an unfamiliar lab while their parents were in another room; during online study visits, children completed testing in their homes while their parents were sitting next to them. While we might expect to see this increased comfort reflected in other tests as well, comfort at the beginning of the study might have differed to a greater extent across the two formats. Information, similarities, and matrix reasoning were administered during the first part of the study visit in both formats. However, in the online format, parents and children played together for 5–10 min prior to the tasks. Although the children were familiarized with the research assistant via play in the lab as well, playing with specifically the parent in the online format could have made children feel more comfortable prior to testing. Finally, it is possible that those who participated online were exposed to factors, whether related to the pandemic or not, that those who participated face-to-face were not. Maybe those who participated online had experiences that benefited their performance on information, similarities, and matrix reasoning. For example, it is possible that those who participated online spent more time interacting with their parents than those who participated face-to-face, due to parents being at home more during the pandemic. Indeed, the verbal skills and acquired knowledge involved in information and similarities, for example, may be sensitive to parental input (Kan et al., 2011; Pace et al., 2016). However, the other tasks included in this study may also benefit from parental input (Clingan-Siverly et al., 2021). Thus, future research should explore whether different types of parental input influenced some cognitive abilities but not others.

Our study has limitations that should be discussed for future studies. First, children may have performed better in the online format due to ordering effects. We did not counterbalance the standardized neurocognitive assessments and experimental tasks for the face-to-face portion of the study. Therefore, we stayed consistent when structing the online portion of the study and continued with the same order of administration. Future research would benefit from counterbalancing the battery of neurocognitive assessments. These neurocognitive assessments demand a lot of attention and children can easily become fatigued throughout the span of assessments. Second, our combined sample was predominately TB children, compared to PTB children. Future research would benefit from having balanced numbers of TB and PTB children. Third, our combined sample was also predominately of a high socioeconomic status. Future research would benefit from having a more diverse sample, so we could better generalize our findings. Last, due to the small sample size, especially in the online group, we may be underpowered to detect main, and more likely, interaction effects. Thus, we argue that our findings should be replicated in future studies with larger samples.

Taken together, our results suggest that online data collection might be a feasible option for several cognitive measures, for both PTB and TB children. Our results also suggest that, however, online data collection for certain measures, including WPPSI-IV information, similarities, and matrix reasoning should be interpreted with caution. Future research should examine the mechanisms through which data collection format might influence children’s performance. Relevant factors to consider include parental involvement and familiarity with the setting. In addition to our empirical findings, we demonstrated success in recruiting and including several families from lower-resourced rural communities and several families with preterm children to participate online, highlighting the feasibility of including samples with different demographic and health characteristics when using online methods. This not only has implications for research methods but also for providing prevention and intervention services. Our results contribute to the growing body of literature examining differences and similarities across various data collection methods. Online data collection may be a good option for continuing developmental psychology research, for diversifying research samples, and for providing services (e.g., educational services and clinical services) when traditional methods are not available. Our findings can also inform future studies hoping to explore the use of online test administration for educational and clinical purposes, as online methods may be more convenient and accessible for both providers and families.

## Data Availability Statement

The raw data supporting the conclusions of this article will be made available by the authors, without undue reservation.

## Ethics Statement

The studies involving human participants were reviewed and approved by University of Iowa Institutional Review Board. Written informed consent to participate in this study was provided by the participants’ legal guardian/next of kin.

## Author Contributions

ÖD-L, PN, and FS conceptualized the study and wrote the manuscript. PN, FS, and HL collected the data. ÖD-L and PN analyzed the data. All authors prepared the data for analysis and contributed to the article and approved the submitted version.

## Conflict of Interest

The authors declare that the research was conducted in the absence of any commercial or financial relationships that could be construed as a potential conflict of interest.

## Publisher’s Note

All claims expressed in this article are solely those of the authors and do not necessarily represent those of their affiliated organizations, or those of the publisher, the editors and the reviewers. Any product that may be evaluated in this article, or claim that may be made by its manufacturer, is not guaranteed or endorsed by the publisher.
